# Dr. Horatio Leseur

**Published:** 1892-10

**Authors:** 


### DR. HORATIO LESEUR.

YOUR committee appointed to prepare resolutions of respect to the memory of the late Dr. Horatio Leseur, of Boston, present the following:

*Resolved*, That we spread upon the records of this Society our appreciation of his long service and uniform genial disposition. Passing from active life at a mature age, he leaves upon the memory of those with whom he has been associated an incentive to be helpful to all around them.

*Resolved*, That this Society tender his family its sincere sympathy in this bereavement.

(Signed) D. B. INGALLS,
S. G. STEVENS,
J. W. BAILEY,
*Committee.*

(Attest) EDGAR O. KINSMAN,
*Secretary.*

